# Habitual Cognitive Reappraisal Was Negatively Related to Perceived Immorality in the Harm and Fairness Domains

**DOI:** 10.3389/fpsyg.2017.01805

**Published:** 2017-10-12

**Authors:** Zhongquan Li, Xiaoyuan Wu, Lisong Zhang, Ziyuan Zhang

**Affiliations:** ^1^School of Social and Behavioral Sciences, Nanjing University, Nanjing, China; ^2^Institute of Disability Research, Nanjing Normal University of Special Education, Nanjing, China; ^3^Department of Applied Foreign Language Studies, Nanjing University, Nanjing, China

**Keywords:** moral judgment, moral foundation theory, emotions, emotion regulation, cognitive reappraisal

## Abstract

Emotion plays an important role in moral judgment, and people always use emotion regulation strategies to modulate emotion, consciously or unconsciously. Previous studies had investigated only the relationship between emotion regulation strategies and moral judgment in the Harm domain, and revealed divergent results. Based on Moral Foundations Theory, the present study extended the investigation into moral judgment in all five moral domains and used a set of standardized moral vignettes. Two hundred and six college students filled in the Emotion Regulation Questionnaire and completed emotional ratings and moral judgment on moral vignettes from Moral Foundations Vignettes. Correlation analysis indicated that habitual cognitive reappraisal was negatively related to immorality rating in Harm, Fairness, and Loyalty domains. Regression analysis revealed that after controlling the effect of other variables, cognitive reappraisal negatively predicted immorality ratings in the Harm and Fairness domains. Further mediation analysis showed that emotional valence only partially explained the association between cognitive reappraisal and moral judgment in Harm area. Some other factors beyond emotional valence were suggested for future studies.

## Introduction

Moral judgment involves assessing the moral acceptability of an action or other characteristics (Avramova and Inbar, 2013; Szekely and Miu, 2015a). Earlier scholars had treated moral judgment mainly as a purely rational process (e.g., Kohlberg, 1971). With the emergence of emotional revolution, however, researchers increasingly recognized the importance of emotion during the process (Greene and Haidt, 2002). Moreover, the proposal of social intuition model (Haidt, 2001) and dual-processing model (Cushman et al., 2010) provided theoretical support for the role of emotion in moral judgment. Cushman et al. (2010) argued that moral judgment was a consequence of interactions between emotion and reason, and Haidt (2001) claimed that moral judgment mainly depended on emotion. Recent studies further revealed that the effect of emotion in moral judgment differed when emotion varied in valence and intensity (Carmona-Perera et al., 2013; Pastötter et al., 2013).

Individuals always regulate their emotions in different ways, either consciously or unconsciously (Gross, 2013b). According to the process model of emotion regulation (Gross, 2013b), emotion regulation can occur at any stage of the emotion-generative process and has effects on emotion, cognition and social behaviors. The process model also distinguishes five families of emotion regulation strategies according to when the strategy acts in the emotion-generative process. Therefore, it is important to consider the role of emotion regulation strategies when discussing the relationship between emotions and moral judgment.

Cognitive reappraisal and expressive suppression are two types of emotion regulation strategies that have been most discussed by scholars (e.g., Ochsner and Gross, 2008). Cognitive reappraisal is an antecedent-focused emotion regulation strategy which involves reframing a situation to change the trajectory of emotional responses, while expressive suppression is a response-focused emotion regulation strategy which involves inhibiting emotion-expressive behavior, such as facial expression. Habitual cognitive reappraisal/expressive suppression refers to a person's disposition in using reappraisal/suppression, and it is often indicated by psychological scales, such as the Emotion Regulation Questionnaire (Gross and John, 2003). In contrast, experimentally induced cognitive reappraisal/expressive suppression often refers to that participants are temporally elicited in using reappraisal/suppression by certain situation or methods (Panno et al., 2013). For example, participants are instructed to use cognitive reappraisal during an experiment session. Although both of these two strategies could effectively reduce the expression of emotions, they differ in their efficacy on emotional experience, physiological response, cognition, and social behaviors (Gross and Levenson, 1997; Richards and Gross, 2000; Gross and John, 2003; Goldin et al., 2008). For instance, cognitive reappraisal was found to be more effective in reducing the experience of negative emotions (Gross, 1998).

Several studies have been conducted to investigate the relationship between these two emotion regulation strategies and moral judgment. Feinberg et al. (2012) found higher habitual application of reappraisal was related to less immoral judgment. They also found emotional intensity significantly mediated the effect of instructed reappraisal and perceptions of immorality. Similarly, Szekely and Miu (2015b) reported that habitual reappraisal negatively predicted deontological choices, and this effect was partly mediated by emotional arousal. Lee and Gino (2015) found that reappraisal had no relationship with moral choices, while suppression resulted in more utilitarian choices. Moreover, they found that deontological inclinations worked as a mediator between suppression and utilitarian decision making. The roles of reappraisal and suppression were quite different in Lee and Gino (2015) and the other two studies. One possible explanation for the divergent findings is that they used different moral dilemmas, and the psychometric properties of these materials had not been examined. In addition, most of those moral dilemmas could be classified into the harm domain, concerning about harming or killing other people.

Moral Foundations Theory (Graham et al., 2011, 2013) broadens the content of moral judgment in exploring moral violations (Simpson and Laham, 2015). It provides a comprehensive framework to understand different domains of morality. According to this theory, there are five moral domains: Harm, Fairness, Authority, Loyalty, and Sanctity. Moral judgment in these five domains involves concerns with suffering of others, concerns with proportional fairness, concerns with deference to authority and tradition, concerns with group loyalty, and concerns with purity and contamination, respectively. These domains exist in most cultures and have different evolutional meanings. Moral judgments in these domains are related to different specific emotions. For example, anger and disgust are related to Harm and Sanctity, respectively (Graham et al., 2013). Recently, Clifford et al. (2015) developed Moral Foundations Vignettes (MFVs) which provided a large set of moral vignettes in these domains, with high reliability and validity.

Valence and arousal are two common dimensional measures of emotion (Posner et al., 2005). Valence assesses the extent to which the emotion experienced is positive or negative, and arousal assesses the extent to which the emotion experienced is intense (Lang, 1985). Emotion regulation strategies are defined to exert influence on emotion, including valence and arousal (Gross, 2013b). Empirical studies indicated that reappraisal leads to higher levels of positive and lower levels of negative emotion experience, while suppression results in decreased levels of positive but not negative emotion experience (e.g., Gross, 2013a). Meanwhile, studies in moral psychology revealed that moral dilemmas eliciting higher emotional arousal or stronger negative valence were more likely to be judged as morally inappropriate (e.g., Greene et al., 2001, 2004; Han et al., 2014). In addition, Szekely and Miu (2015b) directly examined the relationship among cognitive reappraisal, emotional arousal, and moral choices. They reported the partial mediation of emotional arousal on the association between reappraisal and moral choice. That is, reappraisal was negatively related to emotional arousal, which in turn positively predicted deontological moral choice. Therefore, we established a series of mediation models, with emotional valence and arousal as mediators, emotion regulation strategies as independent variables, and moral judgment as dependent variable.

In the present study, we adopted some standardized moral vignettes from Moral Foundations Vignettes and extended the investigation of the association between two habitually used emotion regulation strategies (reappraisal and suppression) and moral judgment to all the five moral domains. We speculated that in general, cognitive reappraisal would predict immorality rating better than expressive suppression due to their effectiveness in regulating negative emotions. That is, the more use of cognitive reappraisal was related to less immorality ratings. However, we could not generate domain-specific hypotheses due to limited literature.

## Materials and methods

### Participants

Totally 206 undergraduate students (49.5% males) at one university participated in our study. Their ages ranged from 17 to 21 years old (*M* = 19.15, *SD* = 0.67). They received course credit as reimbursement.

### Materials

#### Emotion regulation questionnaire (ERQ)

It was developed to assess individual preference for the two emotion regulation strategies, cognitive reappraisal and expressive suppression (Gross and John, 2003). It consists of 10 items, 6 for cognitive reappraisal and 4 for expressive suppression. A sample item in cognitive reappraisal subscale was “when I want to feel less negative emotion (such as sadness or anger), I change what I'm thinking about.),” and a sample item in expressive suppression subscale was “I control my emotion by not expressing them.” Each item is rated on a seven-point Likert-type scale, from 1 (strongly disagree) to 7 (strongly agree). It provides subscale scores for reappraisal and suppression separately. A Chinese revision by Wang et al. (2007) was used in the present study. The Cronbach's *alpha* coefficient for the two subscales was 0.69 and 0.67 in the present sample, respectively.

#### Moral judgment scenarios

We carefully selected 15 scenarios (three for each domain, see **Appendix**) from a standardized and validated collection of moral violating scenarios, Moral Foundations Vignettes (Clifford et al., 2015). The criteria include: (1) large factor loadings; (2) easy to understand. A sample scenario in the Harm domain was “You see a zoo trainer jabbing a dolphin to get it to entertain his customers.” For each scenario, participants evaluated emotional valence, arousal, and immorality on a five-point Likert scale, from 1 (very unpleasant/very calm/no wrongness) to 5 (very pleasant/very intense/very immoral). The Cronbach's *alpha* coefficients ranged from 0.43 to 0.77 for valence rating, from 0.68 to 0.83 for arousal rating, and from 0.60 to 0.78 for immorality rating.

## Results

### Descriptive statistics

Mean, standard deviation, Cronbach's *alpha* and correlations among all the major variables are presented in Table 1. It showed that cognitive reappraisal and expressive suppression was not correlated (*r* = 0.02, *p* > 0.05). Expressive suppression was negatively associated with gender (*r* = −0.22, *p* < 0.01), but the correlation between suppression and immorality ratings didn't reach statistical significance at 0.05 levels (ranged from 0.00 to 0.10, *p* > 0.05). In addition, cognitive reappraisal was significantly related to immorality judgment in the domains of Harm, Fairness, and Loyalty, *r* = −0.34, *p* < 0.01, *r* = −0.25, *p* < 0.01, and *r* = −0.15, *p* < 0.05, respectively.

**Table 1:** Means, standard deviations, reliabilities and correlations for major variables.

	***M***	***SD***	**Skewness**	**Kurtosis**	**1**	**2**	**3**	**4**	**5**	**6**	**7**	**8**	**9**	**10**	**11**	**12**	**13**	**14**	**15**	**16**	**17**	**18**	**19**
1. Age	19.15	0.67																					
2. Gender	0.38	0.49			−0.05																		
3. ERRA	5.23	0.70	−0.40	0.56	0.04	0.07	(0.69)																
4. ERES	3.56	0.97	0.14	−0.39	−0.11	−0.22^**^	0.02	(0.67)															
5. Harm_V	1.68	0.63	1.54	3.41	−0.01	−0.13	0.15^*^	0.07	(0.65)														
6. Fairness_V	1.95	0.59	0.41	0.15	−0.01	0.10	0.10	0.00	0.62^**^	(0.60)													
7. Authority_V	2.25	0.58	−0.17	−0.27	0.11	0.12	−0.02	0.01	0.32^**^	0.51^**^	(0.51)												
8. Loyalty_V	2.69	0.67	−0.16	0.07	−0.05	0.10	0.07	0.05	0.03	0.09	0.24^**^	(0.43)											
9. Sanctity_V	1.72	0.77	1.34	2.24	0.08	−0.08	0.06	0.10	0.57^**^	0.46^**^	0.34^**^	0.08	(0.77)										
10. Harm_A	3.33	0.97	−0.61	0.13	−0.02	0.01	−0.08	0.02	−0.32^**^	−0.11	−0.09	−0.01	−0.28^**^	(0.75)									
11. Fairness_A	2.77	0.89	−0.12	−0.18	0.00	−0.16^*^	−0.09	0.03	−0.11	−0.25^**^	−0.19^**^	−0.02	−0.17^*^	0.68^**^	(0.74)								
12 Authority_A	2.50	0.86	0.06	−0.44	0.01	−0.17^*^	−0.11	−0.11	−0.09	−0.20^**^	−0.24^**^	0.01	−0.21^**^	0.58^**^	0.73^**^	(0.74)							
13. Loyalty_A	2.48	0.86	0.06	−0.67	0.05	−0.07	0.00	−0.16^*^	0.03	−0.12	−0.17^*^	−0.03	−0.19^**^	0.39^**^	0.54^**^	0.65^**^	(0.68)						
14. Sanctity_A	3.21	1.11	−0.45	−0.54	−0.02	−0.09	0.03	−0.02	−0.12	−0.12	−0.06	−0.07	−0.30^**^	0.65^**^	0.62^**^	0.57^**^	0.44^**^	(0.83)					
15. Harm_I	3.73	0.75	−0.54	0.50	0.02	−0.16^*^	−0.34^**^	0.05	−0.22^**^	−0.11	−0.11	0.05	−0.10	0.36^**^	0.29^**^	0.19^**^	0.12	0.16^*^	(0.60)				
16. Fairness_I	3.58	0.79	−0.43	0.28	0.02	−0.20^**^	−0.25^**^	0.04	−0.14^*^	−0.34^**^	−0.21^**^	0.04	−0.15^*^	0.18^**^	0.35^**^	0.24^**^	0.16^*^	0.09	0.65^**^	(0.70)			
17. Authority_I	2.90	0.86	0.10	0.13	−0.04	−0.21^**^	−0.13	0.10	−0.01	−0.15^*^	−0.34^**^	0.02	−0.15^*^	0.03	0.23^**^	0.36^**^	0.29^**^	0.05	0.45^**^	0.58^**^	(0.79)		
18. Loyalty_I	2.16	0.96	0.74	0.11	−0.05	−0.17^*^	−0.15^*^	0.02	0.10	0.00	−0.14^*^	−0.40^**^	−0.09	0.01	0.16^*^	0.28^**^	0.35^**^	0.13	0.27^**^	0.24^**^	0.54^**^	(0.75)	
19. Sanctity_I	3.61	1.10	−0.73	−0.30	0.01	−0.15	−0.07	0.00	−0.09	−0.13	−0.18^**^	−0.09	−0.43^**^	0.19^**^	0.26^**^	0.28^**^	0.21^**^	0.37^**^	0.47^**^	0.45^**^	0.47^**^	0.38^**^	(0.78)

*N = 206*.

* *p < 0.05*,

** *p < 0.01. ERRA, Cognitive Reappraisal; ERES, Expressive Suppression. Harm_V, Harm_A, and Harm_I stand for valence, arousal and immorality rating in Harm area, respectively. The same in other four areas. Cronbach's alphas are shown in parentheses on the diagonal*.

### Regression analysis

In order to have a better understanding of the relations among these major variables, we conducted a series of multiple regression analyses. In each model, moral judgment in certain domain was simultaneously regressed on age, gender, cognitive reappraisal, expressive suppression, emotional valence, and emotional arousal. The detailed results were displayed in Table 2. As can be seen from the Table, after controlling the effects of other variables, emotional valence negatively and emotional arousal positively predicted immorality ratings in all moral domains. However, cognitive reappraisal only negatively predicted immorality ratings in the Harm domain (β = −0.29, *p* < 0.001) and the Fairness domain (β = −0.17, *p* < 0.05). Therefore, in the next section, we mainly focused on investigating the mechanism behind the relationships between cognitive reappraisal and immortality ratings in these two domains.

**Table 2:** Regression analysis on moral judgment in different domains.

**Predictors**	**Harm**	**Fairness**	**Authority**	**Loyalty**	**Sanctity**
Age	0.06	0.03	0.03	−0.07	0.04
Gender	−0.15^*^	−0.13	−0.09	−0.07	−0.16^*^
ERRA	−0.29^***^	−0.17^*^	−0.10	−0.07	0.000
ERES	0.03	−0.02	0.13	0.08	0.012
Valence	−0.20^*^	−0.27^***^	−0.32^***^	−0.37^***^	−0.47^***^
Arousal	0.23^**^	0.20^**^	0.20^**^	0.33^***^	0.17^*^
*R^2^*	0.25	0.21	0.23	0.28	0.31

*N = 206*.

* *p < 0.05*,

** *p < 0.01*,

*** *p < 0.001*.

*ERRA, Cognitive Reappraisal; ERES, Expressive Suppression. Gender: 0 = Male, 1 = Female*.

### Mediational analysis

For these two domains, we first examined the relationship between cognitive reappraisal and immorality ratings, and then explored the mediating roles of emotional valence and emotional arousal. We performed all the above mentioned analyses with bootstrapping PROCESS for SPSS (Hayes, 2013).

**Figure 1:**
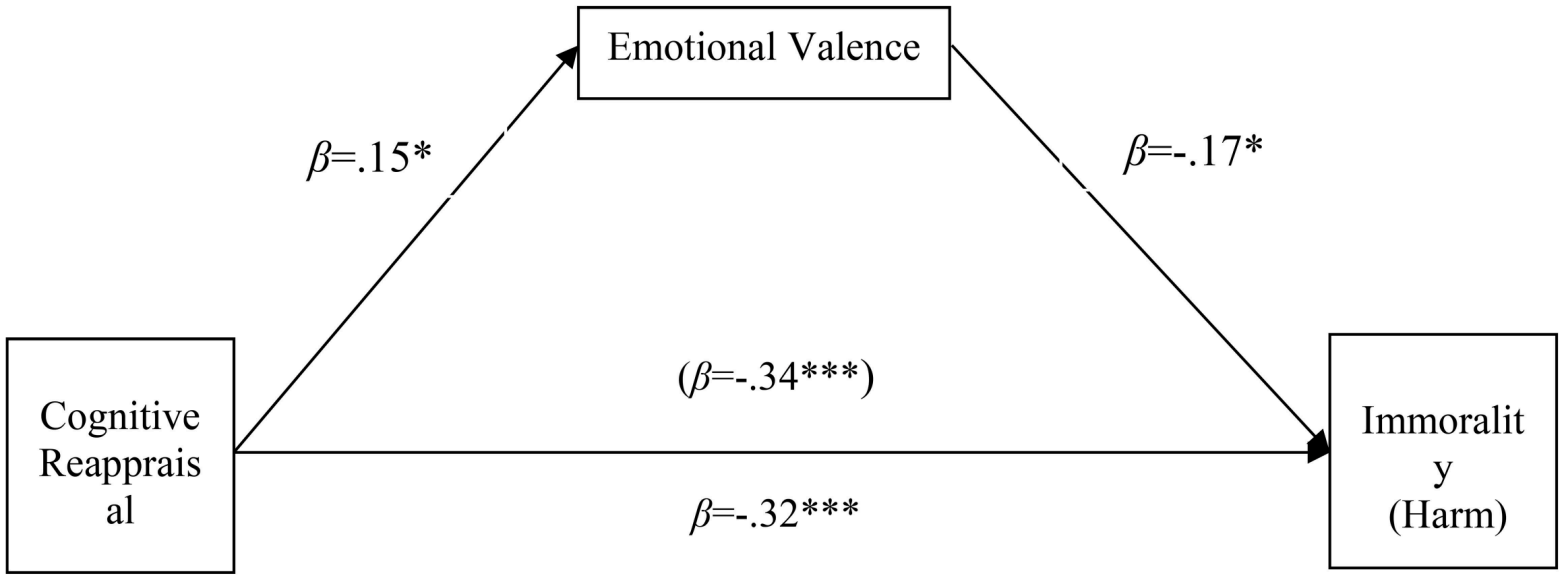
Emotional valence partially mediates the relationship between cognitive reappraisal and morality rating in Harm domain. Standardized regression coefficients are presented. *p < 0.05, **p < 0.01, ***p < 0.001.

In Harm domain, cognitive reappraisal could negatively predict immorality judgment (*b* = −0.37, *SE* = 0.07, *t* = −5.24, *p* < 0.001), indicating that the more often people used habitual cognitive reappraisal, the less immoral they judged. Valence partially mediated the relationship between cognitive reappraisals and moral judgment, and the indirect effect through valence was significant (a1^*^b1 = −0.026; 95% confidence interval [CI] = [−0.071, −0.002]). However, the indirect effect through arousal was not significant (a2^*^b2 = −0.030; 95% confidence interval [CI] = [−0.089, −0.013]). A graphical representation of the results is shown in Figure 1.

In Fairness domain, cognitive reappraisal could negatively predict immorality judgment (*b* = −0.28, *SE* = 0.08, *t* = −3.65, *p* = 0.00). However, the indirect effect through neither valence nor arousal was significant (a1^*^b1 = −0.035, 95% CI = [−0.0959, 0.0136]; a2^*^b2 = −0.0328; 95% CI = [−0.0958, 0.0199]).

## Discussion

Emotion plays an important role in moral judgment, and people always use emotion regulation strategies to modulate emotion, consciously or unconsciously. This study explored the association of two emotion regulation strategies (i.e., cognitive reappraisal and expressive suppression) with moral judgment in different domains. Results indicated that compared with expressive suppression, cognitive reappraisal had a closer relationship with moral judgment. The associations between cognitive reappraisal and moral judgment in Harm, Fairness, and Loyalty domains were significant, while expressive suppression was not significantly related to moral judgment in any of the five moral domains. Results also showed that emotional valence only partially mediated the association between reappraisal and morality judgment in Harm domain. When facing moral judgment scenarios in Harm domain, individuals who inclined to use cognitive reappraisal could reduce their emotional valence more effectively and provide lower levels of immorality judgment. The results partly supported our hypothesis about the relationship between emotion regulation strategies and moral judgment.

Previous studies investigating the relationship between emotion regulation strategies and moral judgment in the harm domain revealed divergent results. The present study addressed the divergence with a set of standardized moral scenarios. Both Feinberg et al. (2012) and Szekely and Miu (2015b) found that only cognitive reappraisal was related to moral judgment, and higher cognitive reappraisal was significantly associated with less immoral judgment. Nevertheless, Lee and Gino (2015) found only expressive suppression was related to moral judgment, and the higher the expressive suppression was, the more the utilitarian choices would be. Our result was consistent with findings in both Feinberg et al. (2012) and Szekely and Miu's (2015b). In addition, emotional valence only partially accounted for the association between cognitive reappraisal and moral judgment in Harm area. Compared with expressive suppression, cognitive reappraisal is more effective in regulating negative emotions, therefore it leads to decreased immoral judgment and increased deontological choices. More negative emotion valence was related to higher immorality rating, which was in agreement with previous studies (e.g., Greene et al., 2001, 2004; Han et al., 2014). Due to partial meditation, there should be some other factors beyond the emotional valence for the association between cognitive reappraisal and moral judgment. One possible candidate is cognitive resource limitation. Cognitive reappraisal occupies less cognitive resources when regulating negative emotions (Richards and Gross, 2000, 2006), and therefore leaves more space for utilitarian decisions or adjustment from emotion-driven deontological decisions, which involves more executive resources (Moore et al., 2008).

We also extended the current investigation of the associations between cognitive reappraisal and moral judgment to the other four moral domains suggested by moral foundations theory. The results indicated that the associations were significant in Fairness, and Loyalty domains. The different relationship patterns might be attributed to the different types of emotion induced in these moral scenarios (Zhang et al., 2017). That is, moral scenarios “vary systematically in the extent to which they engage emotional processing and that these variations in emotional engagement influence moral judgment” (Greene et al., 2001, p. 2105). Moral scenarios in Harm, Fairness, and Loyalty areas mainly elicit anger at perpetrator, cheater, or traitors, while moral scenarios in Sanctity area only elicit disgust. However, whether moral scenarios in Authority area may induce somewhat anger at violator depends on the subcultures, social conservatives or social liberals (Graham et al., 2013). The different relationship patterns might be also attributed to the differences in valence and arousal induced by moral vignettes. Moral scenarios in the Harm domain are classified as “high-conflict” and induce the most intense emotions (Szekely and Miu, 2015b). We conducted further comparisons of valence and arousal in different moral domains, and found emotional valence in the Harm domain was significantly more negative than that in the Fairness, Authority, and Loyalty areas, and emotional arousal in the harm domain was significantly higher than that in all the other four domains.

Several limitations should be mentioned here. One limitation is that due to the difference in informativeness and vividness, although moral vignettes in the present study have been standardized with sound psychometric properties, they may not induce emotions as intense as those in previous studies using typical moral dilemmas. To overcome this limitation, some new techniques such as virtual reality can be introduced to increase the realism of moral judgment (e.g., Patil et al., 2014). Another limitation is that the method of measuring emotional valence and arousal is self-reported by participants. To overcome this limitation, some objective indexes such as physiological measures can be included to provide more information about emotions during the process of moral judgment (e.g., Stellar et al., 2015). The third, recent studies indicated that moral judgment was related to autobiographical memory processing, and shared several common brain regions (e.g., the default mode network) with self-related processes (Han et al., 2016; Han, 2017; Knez and Nordhall, 2017). We didn't consider the potential involvement of self-related processes, such as self-regulation, in moral judgment here. Further studies may need to examine the role of self-related processes in the association between emotion regulation strategies and moral judgment. Fourthly, the cultural background should be introduced in comparing the differences in moral judgment between participants from Eastern cultures and from Western cultures. In addition, the lack of controlling variables, such as socio-demographics, affective state, limits the generalizability of findings in the present study.

In sum, the present study extended our understanding of the relationship between emotion regulation strategies and moral judgments in two ways: (1) addressing previous divergence in the harm domain with a set of standardized moral vignettes; (2) providing evidence for the association between emotion regulation strategies and moral judgment in the other four domains. The study also indicated the role of emotional route (i.e., via emotional valence and arousal) in the relationship. Although emotion plays an important role in moral judgment process, it is not the whole story of moral judgment. There should be some other factors beyond emotional valence to explain the relationship. Further studies are needed to examine such other factors as cognitive resource limitation.

## Ethics statement

The study protocol was approved by the ethics committee of Department of Psychology, Nanjing University. The study was carried out in accordance with the guidelines of the Helsinki Declaration with written informed consent from all subjects.

## Author contributions

All authors conceived, designed and conducted the studies, ZL conducted the statistical analyses, ZL and XW wrote the first draft, and all authors revised the final manuscript.

### Conflict of interest statement

The authors declare that the research was conducted in the absence of any commercial or financial relationships that could be construed as a potential conflict of interest.

## Appendix

### Moral judgment scenarios

**Table:**

**No**.	**Domain**	**Scenario**
1	Harm	You see a zoo trainer jabbing a dolphin to get it to entertain his customers.
2	Harm	You see a man lashing his pony with a whip for breaking loose from its pen.
3	Harm	You see a woman spanking her child with a spatula for getting bad grades in school.
4	Fairness	You see an employee lying about how many hours she worked during the week.
5	Fairness	You see someone cheating in a card game while playing with a group of strangers.
6	Fairness	You see a runner taking a shortcut on the course during the marathon in order to win.
7	Authority	You see a man turn his back and walk away while his boss questions his work.
8	Authority	You see a star player ignoring her coach's order to come to the bench during a game.
9	Authority	You see a teaching assistant talking back to the teacher in front of the classroom.
10	Loyalty	You see a coach celebrating with the opposing team's players who just won the game.
11	Loyalty	You see the class president saying on TV that her rival college is a better school.
12	Loyalty	You see a teacher publicly saying she hopes another school wins the math contest.
13	Sanctity	You see a drunk elderly man offering to have oral sex with anyone in the bar.
14	Sanctity	You see a man searching through the trash to find women's discarded underwear.
15	Sanctity	You see a man in a bar using his phone to watch people having sex with animals.
